# Simultaneous Quantification of Pioglitazone and Omarigliptin in Rat Plasma by UHPLC-MS/MS and Its Application to Pharmacokinetic Study after Coadministration of the Two Drugs

**DOI:** 10.1155/2021/6693366

**Published:** 2021-06-07

**Authors:** Lin Wang, Jiaxi Wang, Chao Lin, Furong Wang, Xiangping Li, Wanhui Liu

**Affiliations:** ^1^School of Pharmacy, Key Laboratory of Molecular Pharmacology and Drug Evaluation (Yantai University), Ministry of Education, Collaborative Innovation Center of Advanced Drug Delivery System and Biotech Drugs in Universities of Shandong, Yantai University, Yantai 264005, China; ^2^Yantai Key Laboratory of Nanomedicine & Advanced Preparations, Yantai Institute of Materia Medica, Yantai 264000, China; ^3^ShanDong Luye Pharmaceut Co Ltd, Yantai 264000, Shandong, China

## Abstract

Combination therapy is a common approach for clinical treatment of type 2 diabetes mellitus, especially for patients with poor monotherapy. Meta-analysis suggested that omarigliptin, a long-acting DPP-4 inhibitor, combined with pioglitazone might improve the side effects of pioglitazone. However, little is known about the pharmacokinetic properties after a coadministration. In this study, a rapid and reliable method for the simultaneous determination of the pioglitazone and omarigliptin in rat plasma by UHPLC-MS/MS was established and validated for the first time. An exsil mono C18 column (2.0 × 50 mm, 3 *μ*m) was used to separate the analytes and the column temperature was kept at 30°C. Sitagliptin was selected as the internal standard. 0.02% formic acid aqueous solution (A) and methanol-acetonitrile (B) were used as mobile phases with gradient elution at a flow rate of 0.3 mL/min. The elution procedure was as follows: 20%B (0–0.1 min), 80%B (0.1–0.3 min), 80%B (0.3–2.0 min), and 20%B (2.1–3.0 min). A multiple reaction monitor (MRM) was used under positive ionization mode with electrospray ion source to detect pioglitazone (357.1 ⟶ 134.1), omarigliptin (399.2 ⟶ 153.0), and sitagliptin (408.2 ⟶ 235.0). The linear ranges of pioglitazone and omarigliptin were 5–2000 ng/mL and 10–4000 ng/mL, respectively. Good linear relationships were exhibited in the corresponding linear ranges (*r* ≥ 0.9944). The bioanalytical method was validated, and the selectivity, linearity, sensitivity, accuracy, precision, stability, recovery, and matrix effect were acceptable. The validated method was then successfully applied to pharmacokinetic study of pioglitazone combined with omarigliptin in rats. Results suggested that the combination of the two drugs had little effect on the pharmacokinetic parameters of each other in rats.

## 1. Introduction

Type 2 diabetes mellitus (T2DM) is one of the most common metabolic diseases around the whole world. It is mainly caused by pancreatic *ß* cell dysfunction and insulin resistance, leading to continuous increase in blood glucose [1]. Statistics demonstrated that more than 415 million adults suffered from T2DM [2, 3]. There are many treatments for T2DM, such as exercise, surgery, and traditional Chinese medicine and drug therapy. Among them, oral hypoglycemic drug treatment is the most convenient and effective method.

Pioglitazone, a member of the thiazolidinediones, was an oral antidiabetic drug widely used in China [4]. It is an insulin sensitizer that can reduce insulin resistance in peripheral tissues and liver, and meanwhile increase the uptake of peripheral glucose by activating peroxisome proliferator-activated receptor gamma (PPAR *γ*). Therefore, blood glucose could be controlled but the endogenous secretion of insulin has not been increased yet [5]. Studies have shown that pioglitazone might reduce major adverse cardiovascular events, correct multiple components of metabolic syndrome, and improve nonalcoholic fatty liver disease [6, 7]. However, side effects such as weight gain, fluid retention, or even fracture could not be ignored either [7, 8].

Dipeptidyl peptidase-4 (DPP-4) inhibitors could improve glycemic control in patients with T2DM and have gained extensive interest because of long-term efficacy and better glycemic control. DPP-4 inhibitors exerted a hypoglycemic effect by inhibiting the inactivation of glucagon-like peptide-1 (GLP-1), promoting the release of insulin from pancreatic *ß* cells and inhibiting the secretion of glucagon from pancreatic *α* cells [9, 10]. Furthermore, there were some other advantages for DPP-4 inhibitors like not increasing the weight, decreasing the risk of hypoglycemia, repairing the function of pancreatic islet, and protecting the cardiovascular system [11]. More importantly, recent studies have found that DPP-4 inhibitors may exhibit potent neuro-restorative effects [12]. Consequently, DPP-4 inhibitors have been gradually used in clinical practice.

When the blood glucose could not be controlled by monotherapy any more, the combination should be considered. Studies have suggested that the combination of DPP-4 inhibitors and thiazolidinediones could simultaneously solve insulin resistance and islet dysfunction, two major defects of T2DM [13]. In addition, DPP-4 inhibitors could enhance the excretion of sodium and fluid in kidney, thus alleviating the possible side effects of pioglitazone [14]. Moreover, the combination therapy could significantly improve the function of pancreatic *ß* cells and had a better effect on blood glucose control [15]. Meta-analysis showed that, compared with pioglitazone alone, the combination could remarkably reduce hemoglobin A1c (HbA1c) levels and fasting blood glucose [16]. Therefore, the combination therapy is considered more suitable for patients with poor curative effect by monotherapy. Omarigliptin, a weekly DPP-4 inhibitor, which has been approved in Japan since 2015, showed more effective than sitagliptin [17]. Compared with the once-daily administered DPP-4 inhibitors available in market, omarigliptin significantly improved the compliance of patients. Although a micelle-enhanced spectrofluorimetric method was developed for determination of omarigliptin [18], a HPLC method was used to investigate the degradation kinetics of omarigliptin in the oxidative and photolytic medium [19], and the literature has revealed pharmacokinetics of omarigliptin [20], there is no study to elucidate the pharmacokinetic or pharmacodynamic characteristics of the combination of omarigliptin and pioglitazone.

The aim of this study was to investigate the pharmacokinetic characteristics of the omarigliptin combined with and pioglitazone in rats. For this purpose, a simple and sensitive method for the simultaneous quantification of pioglitazone and omarigliptin in rat plasma needed to be established first. Then, the validated method was successfully applied to the pharmacokinetic study of pioglitazone combined with omarigliptin in rats. To the best of our knowledge, there was no similar study before, and the results may give hints for the combined administration of pioglitazone and omarigliptin.

## 2. Materials and Methods

### 2.1. Chemicals and Reagents

**Figure 1:**
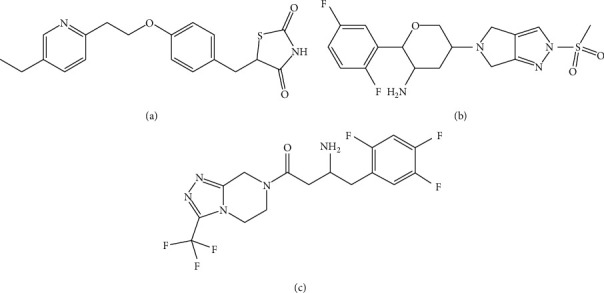
Structures of pioglitazone (a), omarigliptin (b), and sitagliptin (c).

Pioglitazone hydrochloride (pharmaceutical secondary standard) was purchased from Merck Life Science Co. Ltd. (Shanghai, China). Omarigliptin (purity>98%) (Batch Number: 20190709) was synthesized by Dr Lin (Yantai Institute of Materia Medica). Sitagliptin (internal standard, purity>98%) was purchased from Bide Pharmatech Ltd. (Shanghai, China). Structures of pioglitazone, omarigliptin, and sitagliptin are shown in Figure 1. Formic acid (FA) was bought from Fluka Chemie (Buchs, Switzerland). Methanol and acetonitrile were HPLC grade and purchased from Fisher Scientific (Pittsburgh, USA). Deionized water was manufactured by Milli-Q purification system (Bedford, USA).

### 2.2. Instrument and Conditions

Dionex Ultimate 3000 UHPLC system including two RS pumps, an RS autosampler, and an RS column compartment was employed in this study (Thermo Scientific, Massachusetts, USA). An Exsil Mono C18 column (2.0 × 50 mm, 3 *μ*m, Dr. Maisch GmbH, Ammerbuch-Entringen, Germany) was used to achieve the chromatographic separation. Deionized water containing 0.02% (v/v) formic acid was labelled as mobile phase A and methanol: acetonitrile (v/v, 1 : 1) was labelled as mobile phase B. Gradient elution program is listed in Table 1. The column temperature was held at 30°C and the autosampler temperature was kept at 10°C. The injection volume was 2 *μ*L.

TSQ Quantiva triple quadrupole mass spectrometer with Xcalibur (Thermo Scientific, Massachusetts, USA) was adopted for precise quantification. Data were acquired under positive ionization mode. Individual parameters for the analytes and IS such as ion pairs, collision energies, and RF lens are displayed in Table 2. Other mass spectrometry parameters were set as follows: positive ion, 3500 V; ion transfer tube temperature, 350°C; vaporizer temperature, 300°C; sheath gas, 35 Arb; aux gas, 15 Arb; sweep gas, 2 Arb; CID gas, 1.5 mTorr. Dwell times were 100 msec for all compounds.

### 2.3. Preparation of Standard Calibration Samples and Quality Control Samples

All compounds were dissolved and diluted with methanol to prepare stock solutions and working solutions. All solutions were stored at 4°C.

Different concentrations of working solutions were diluted 20 times with blank rat plasma to prepare corresponding calibration samples. Final concentrations of calibration samples were 5, 25, 50, 100, 250, 500, 1000, and 2000 ng/mL for pioglitazone and 10, 50, 100, 200, 500, 1000, 2000, and 4000 ng/mL for omarigliptin. Quality control (QC) samples (5, 15, 400, 1500 ng/mL for pioglitazone and 10, 30, 800, 3000 ng/mL for omarigliptin) were prepared as the same way. All plasma samples were stored at −20°C until analysis.

### 2.4. Extraction of Plasma Samples

Whole blood was centrifuged at 8000 rpm for 5 min to obtain plasma samples, which were then extracted by simple protein precipitation. In brief, 25 *μ*L of thawed plasma was added to 800 *μ*L IS solution (sitagliptin, 50 ng/mL) and was whirled for 1 min to mix well. Centrifugation was performed at 12000 rpm for 5 min and then supernatant fluid was obtained for analysis.

### 2.5. Method Validation

The method was validated according to the US Food and Drug Administration (FDA) guidelines [21].

#### 2.5.1. Selectivity

The selectivity was evaluated by comparing blank plasma from six different rats, blank plasma spiked with analytes and IS, and practical plasma samples. The intensity of blank samples should be less than 20% of LLOQ samples.

#### 2.5.2. Linearity and Sensitivity

The calibration curve was fitted by plotting the peak area ratio of the analyte to IS against the nominal concentration of the analyte in plasma, and the weighting coefficient was set to 1/*x*^2^. The minimum concentration of the standard curve was taken as the lower limit of quantification (LLOQ). The precision and accuracy of LLOQ should be 80–120%.

#### 2.5.3. Precision and Accuracy

The intraday precision and accuracy of the method were evaluated by analyzing QC samples at low, medium, and high concentrations (*n* = 6) on the same day. Results of three consecutive days were used to assess the interday precision and accuracy. Precision and accuracy were characterized by relative standard deviation (RSD, %) and relative error (RE, %), respectively. RE (%) and RSD (%) should be within ±15%.

#### 2.5.4. Recovery and Matrix Effect

In order to calculate the extraction recovery and matrix effect, blank plasma samples were collected from six different rats and processed as follows. (A) Blank plasma samples were spiked with analytes and then were precipitated with methanol containing IS. (B) The blank plasma was precipitated with pure methanol to obtain supernatant, and then analytes and IS with the same concentration as group A were added to the supernatant. (C) The working solution was added to an equal amount of deionized water instead of blank plasma and then were extracted with methanol containing IS as described in Section 2.4. The recovery was calculated by dividing the peak area of group A by that of group B, while the matrix effect was calculated by dividing the peak area of group B by that of group C.

#### 2.5.5. Stability

QC samples with low, medium, and high concentrations were analyzed under different conditions, such as kept at room temperature for 6 h, frozen and thawed for three cycles, and frozen at −20°C for 7 days. The postextraction samples were stored in autosampler at 10°C for 24 hours and then reinjected to evaluate the autosampler stability. The measured concentrations were calculated according to the following standard curve. RE (%) and RSD (%) should be within ±15%.

#### 2.5.6. Diluted Quality Control Samples

Diluted QC samples were prepared by diluting a high concentration of QC samples with blank rat plasma and six diluted QC samples were prepared in parallel. The concentration was calculated based on the accompanying calibration curve.

### 2.6. Pharmacokinetics Study

Fifteen male Sprague-Dawley rats (6–8 weeks old, 190–210 g) were purchased from Jinan Pengyue Laboratory Animal Technology Co., Ltd. (Jinan, China). The animal certification number was 3707262011004044 and the production license number was SCXK (Lu) 20190003. Before the formal experiment, all rats were adaptively fed for one week at a temperature of 24 ± 2°C and a humidity of 55 ± 5% and then were randomly divided into three groups. In order to guarantee animal welfare, Animal Experiments Guidelines of Yantai Institute of Materia Medica (Yantai, China) was enforced.

Pioglitazone and omarigliptin were suspended into 0.5% carboxymethyl cellulose sodium salt (CMC-Na) for intragastric administration. The dosage for rats was calculated based on the clinical dose. Rats were fasted for at least 12 h before the experiment to avoid possible effects of food and then were administrated with pioglitazone (2.6 mg/kg), omarigliptin (2.6 mg/kg), or a combination of the two compounds, respectively. About 200 *μ*L whole blood was collected into the heparinized tube at 0 min (pre-dose), 15 min, 30 min, 1 h, 2 h, 4 h, 6 h, 8 h, 12 h, 24 h, and 48 h and centrifugated immediately to collect plasma. All plasma samples were frozen at −20°C until analysis. Samples were processed and analyzed by the validated method.

### 2.7. Data Analysis

Data were acquired and analyzed by Xcalibur 4.1 software (Thermo Fisher Scientific, Massachusetts, USA). Pharmacokinetic parameters were calculated based on noncompartmental model using Phoenix WinNonlin 7.0 (Pharsight, Mountain View, CA). All data were shown as mean ± SD.

## 3. Results and Discussion

### 3.1. Method Development and Optimization

**Figure 2:**
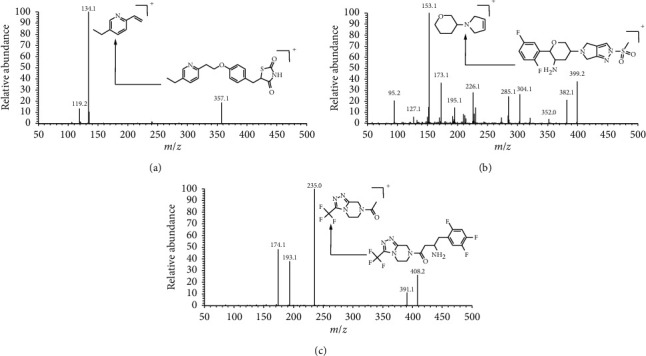
The product ion mass spectra of pioglitazone (a), omarigliptin (b), and sitagliptin (c).

Full mass scan was performed under positive mode and [M + H]^+^ were formed for these three compounds probably due to the presence of amino groups. The corresponding product ions were then confirmed and relevant mass parameters such as collision energy and RF lens were optimized for higher response (Figure 2, Table 2). A satisfactory resolution was obtained on an Exsil Mono C18 column (2.0 × 50 mm, 3 *μ*m) with gradient elution. In order to increase the response, 0.02% formic acid was added to the aqueous phase. Methanol: acetonitrile (v/v, 1 : 1) was used to improve the shape of chromatographic peak instead of pure methanol. The samples were extracted by protein precipitation. Compared with the liquid-liquid extraction methods reported in the literature to extract omarigliptin from biological samples [22, 23], the protein precipitation was simple, cheap, and time-saving.

### 3.2. Method Validation

#### 3.2.1. Selectivity

**Figure 3:**
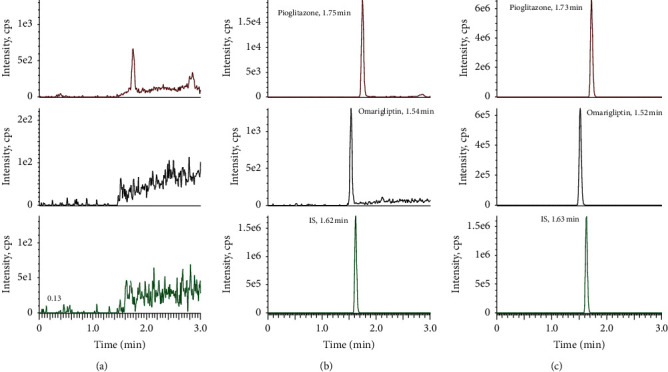
Representative MRM chromatograms for pioglitazone, omarigliptin, and IS from (a) blank rat plasma; (b) blank plasma spiked with pioglitazone (5 ng/mL), omarigliptin (10 ng/mL), and IS (50 ng/mL); and (c) plasma from rats at 2 h after oral administration of pioglitazone and omarigliptin.

As shown in Figure 3, there was no obvious endogenous interference and the retention times for pioglitazone, omarigliptin, and sitagliptin (IS) were 1.75 min, 1.54 min, and 1.62 min, respectively. The peak shape and resolution were good and the selectivity of this method was satisfactory.

#### 3.2.2. Linearity and Sensitivity

**Figure 4:**
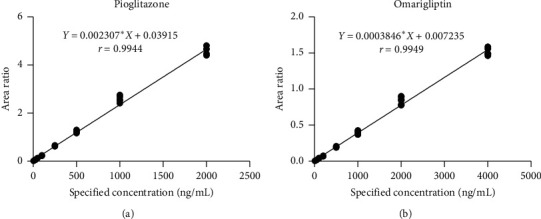
Calibration curves for pioglitazone (5–2000 ng/mL) and omarigliptin (10–4000 ng/mL) over 3 days.

Linear regression was performed with the concentration as the abscissa and the peak area ratio of the analyte to IS as the ordinate. The standard curve equation was calculated by least square method with weighting factor (*w* = 1/*x*^2^). As shown in Figure 4, the pioglitazone and omarigliptin exhibited good linearity during their respective linear ranges. The sensitivity is shown in Table 3 and the results were acceptable.

#### 3.2.3. Precision and Accuracy

Table 3 indicates the precision and accuracy of the method. The intraday precision (RSD, %) of pioglitazone and omarigliptin was less than 7.6% and the accuracy (RE, %) was within ±8.7%. The interday precision (RSD, %) was less than 6.9% and accuracy (RE, %) was within ±8.4%. The method was accurate and reliable.

#### 3.2.4. Recovery and Matrix Effect

As shown in Table 4, the recoveries of pioglitazone and omarigliptin at three concentrations were 86.7%–96.5% and 89.1%–94.8% and the recovery of IS was 107%. The recoveries were satisfactory. Meanwhile, the RSD of all QC samples prepared with biological matrix from different sources was less than 7.7% (Table 4), indicating that the method was reproducible, so the matrix effect was tolerable.

#### 3.2.5. Stability

The results are shown in Table 5. RE and RSD were less than 15% under different conditions, demonstrating the stability of the method was acceptable.

#### 3.2.6. Diluted Quality Control Samples

Since the concentration of individual samples exceeded the upper limit of quantification, appropriate dilution was required before detection. So it was necessary to study diluted quality control samples. QC samples containing 3000 ng/mL pioglitazone and 6000 ng/mL omarigliptin were diluted twice with blank plasma and then precipitated and centrifuged for injection analysis.  Table 6 shows that both RSD and RE were less than 15%, and the diluted QC samples were valid.

### 3.3. Pharmacokinetics Study

**Figure 5:**
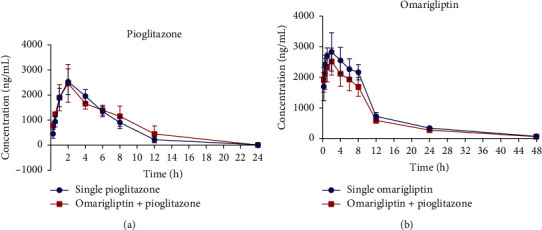
Mean plasma concentration-time curves after oral administration of pioglitazone (2.6 mg/kg), omarigliptin (2.6 mg/kg), or pioglitazone (2.6 mg/kg) plus omarigliptin (2.6 mg/kg) (mean ± SD, *n* = 5).

The pharmacokinetic parameters after single or combined administration are displayed in Tables 7 and 8.  Figure 5 shows the mean plasma concentration-time curves for pioglitazone and omarigliptin. Both pioglitazone and omarigliptin were absorbed rapidly after a single administration, with *t*_max_ being 1.8 h and 1.5 h, respectively. The elimination of omarigliptin was much slower than that of pioglitazone, with t1/2 of omarigliptin being 10.9 h while *t*1/2 of pioglitazone was 2.8 h. The AUC _(0-t)_ and AUC _(0-∞)_ were 16887 and 16973 ngh/mL for pioglitazone alone, and there was a slight increase after combining with omarigliptin, but with no statistical significance (18988 and 19013 ngh/mL). On the contrary, after coadministration, the bioavailability of omarigliptin decreased gently (Table 8). In general, the combination had little effect on pharmacokinetic parameters compared with single administration. It was worth noting that the pharmacokinetic study was only investigated on normal rats in this paper; further studies should be carried out on diabetic models to explore the pharmacokinetic and pharmacodynamic characteristics to ensure the effectiveness and safety of the combination therapy.

## 4. Conclusion

Drug combination is commonly used clinically to treat patients with T2DM who are not sensitive to monotherapy. Pioglitazone combined with omarigliptin could simultaneously solve insulin resistance and islet dysfunction in patients with T2DM, which can be used as an option for treatment. In this study, the quantification and pharmacokinetics of the two drugs were reported. Results indicated that the pharmacokinetic behavior of pioglitazone and omarigliptin was almost unchanged after the combined administration. From the perspective of PK, there was no obvious interaction between the two drugs, suggesting that there may be no need to adjust the dose when used together. The research provided an experimental basis for evaluating the efficacy and safety of pioglitazone combined with omarigliptin in vivo and laid a foundation for wider clinical applications.

## Figures and Tables

**Table 1:** Gradient elution procedure of the method.

Time (min)	Flow rate (mL/min)	A (%)	B (%)
0	0.3	80	20
0.1	0.3	80	20
0.3	0.3	20	80
2.0	0.3	20	80
2.1	0.3	20	20
3.0	Stop		

**Table 2:** Optimized parameters for the detection of pioglitazone, omarigliptin, and sitagliptin (IS).

Compounds	Precursor ions	Product ions	CE (V)	RF lens (V)
Pioglitazone	357.1	134.1	29	100
Omarigliptin	399.2	153.0	30	80
Sitagliptin	408.2	235.0	18	80

**Table 3:** Precision and accuracy for the analysis of pioglitazone and omarigliptin in rat plasma (*n* = 6).

		Intraday			Interday		
Compound	Nominal conc.	Measured conc.	Precision	Accuracy	Measured conc.	Precision	Accuracy
	(ng/mL)	(Mean ± SD)	(RSD, %)	(RE, %)	(Mean ± SD)	(RSD, %)	(RE, %)
*Pioglitazone*	5	5.03 ± 0.09	1.76	−1.59	5.40 ± 0.47	8.62	7.96
	15	15.39 ± 1.17	7.59	−4.84	15.61 ± 1.08	6.93	4.06
	400	366.39 ± 1.36	0.37	−8.68	366.59 ± 1.18	0.32	−8.35
	1500	1439.91 ± 4.68	0.33	−4.09	1437.11 ± 5.49	0.38	−4.19

*Omarigliptin*	10	9.92 ± 0.17	1.75	−0.77	10.51 ± 0.71	6.74	5.05
	30	32.00 ± 0.41	1.28	6.67	30.60 ± 1.69	5.53	2.00
	800	734.28 ± 2.88	0.39	−8.21	733.20 ± 9.05	1.23	−8.35
	3000	2947.16 ± 10.01	0.34	−1.76	2946.69 ± 12.01	0.41	−1.78

**Table 4:** Recovery and matrix effects of pioglitazone and omarigliptin in rat plasma (*n* = 6).

Analytes	Concentration	Recovery (%)		Matrix effect (%)	
	(ng/mL)	Mean ± SD	RSD (%)	(Mean ± SD)	RSD (%)
*Pioglitazone*	15	94.56 ± 5.79	6.13	158.98 ± 9.63	6.06
	400	96.45 ± 6.59	6.83	134.90 ± 9.56	7.09
	1500	86.73 ± 5.38	6.20	142.64 ± 8.60	6.03

*Omarigliptin*	30	94.79 ± 4.59	4.72	113.80 ± 5.40	4.74
	800	96.42 ± 6.19	6.42	108.91 ± 6.89	6.32
	3000	89.11 ± 7.09	7.96	111.84 ± 8.59	7.68

*Sitagliptin*	50	107.01 ± 2.08	2.04	98.00 ± 2.05	2.09

**Table 5:** Stabilities of pioglitazone and omarigliptin under different conditions (*n* = 5).

Storage conditions	Compound	Nominal concentration (ng/mL)	Measured concentrations (ng/mL)	Precision (RSD %)	Accuracy (RE %)
*Room temperature for* 6 h	Pioglitazone	15	14.31 ± 0.34	2.38	−4.62
		400	361.75 ± 1.91	0.53	−9.56
		1500	1418.82 ± 5.26	0.37	−5.41
	Omarigliptin	30	30.01 ± 0.40	1.33	0.05
		800	738.06 ± 4.52	0.61	−7.74
		3000	2937.89 ± 7.68	0.26	−2.07

*Three freeze-thaw cycles*	Pioglitazone	15	13.78 ± 0.11	0.81	−8.12
		400	402.08 ± 1.11	0.28	0.52
		1500	1333.57 ± 7.61	0.57	−11.10
	Omarigliptin	30	28.46 ± 0.25	0.86	−5.13
		800	794.54 ± 9.37	1.18	−0.68
		3000	2670.56 ± 16.54	0.62	−10.98

*Auto-sampler stability* (10°C *for* 24 h)	Pioglitazone	15	15.59 ± 1.05	6.72	3.95
		400	363.31 ± 1.73	0.48	−9.17
		1500	1421.47 ± 7.30	0.51	−5.24
	Omarigliptin	30	32.01 ± 2.13	6.64	6.70
		800	762.47 ± 4.84	0.63	−4.69
		3000	2997.38 ± 9.39	0.31	−0.09

*Freeze* (−20*°C for* 7 *days*)	Pioglitazone	15	13.68 ± 0.10	0.75	−8.77
		400	400.95 ± 1.64	0.41	0.24
		1500	1328.31 ± 5.99	0.45	11.45
	Omarigliptin	30	28.56 ± 0.11	0.40	−4.79
		800	802.26 ± 4.01	0.50	0.28
		3000	2689.12 ± 45.57	1.69	−10.36

**Table 6:** Precision and accuracy for the diluted QC samples (*n* = 6).

Compound	Spiked concentration	Measured concentration	Precision	Accuracy
	(ng/mL)	(Mean ± SD)	(RSD, %)	(RE, %)
Pioglitazone	3000	3248.87 ± 470.52	14.48	8.30
Omarigliptin	6000	6734.21 ± 907.56	13.48	12.24

**Table 7:** Pharmacokinetic parameters of pioglitazone in rats after single or combination administration (*n* = 5).

	*C*_max_	*t*_max_	*t*_1/2_	AUC_(0−t)_	AUC_(0−∞)_
	(ng/mL)	(hour)	(hour)	ng·h/mL	ng·h/mL
Single pioglitazone	2551 ± 475	1.8 ± 0.4	2.8 ± 1.0	16887 ± 2209	16973 ± 2127
Combination	2465 ± 754	2.0 ± 0	2.1 ± 0.2	18988 ± 2582	19013 ± 2604

**Table 8:** Pharmacokinetic parameters of omarigliptin in rats after single or combination administration (*n* = 5).

	*C*_max_	*t*_max_	*t*_1/2_	AUC_(0−t)_	AUC_(0−∞)_
	(ng/mL)	(hour)	(hour)	ng·h/mL	ng·h/mL
Single omarigliptin	2997 ± 394	1.5 ± 0.7	10.9 ± 0.5	36551 ± 4065	37699 ± 4101
Combination	2521 ± 435	1.8 ± 0.4	11.0 ± 1.2	30357 ± 3762^*∗*^	31346 ± 3815^*∗*^

## Data Availability

The data used to support the findings of this study are available from the corresponding author upon request.
